# Supplementary figures and images for: Ixodes scapularis Is the Most Susceptible of the Three Canonical Human-Biting Tick Species of North America to Repellent and Acaricidal Effects of the Natural Sesquiterpene, (+)-Nootkatone
Source: Insects. 2023 Dec 22;15(1):8. doi: 10.3390/insects15010008 (PMC10816182; doi:10.3390/insects15010008)

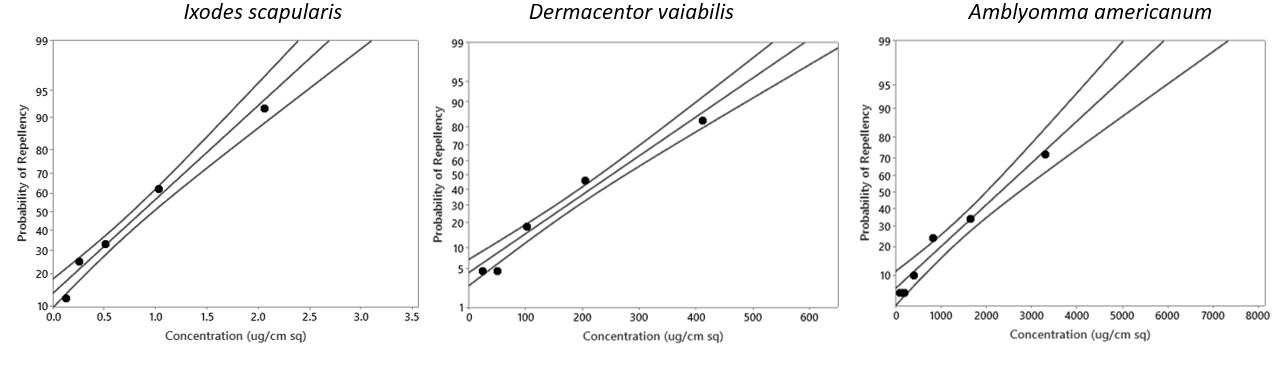

Supplement: Supplementary file 1 [file insects-15-00008-s001.zip › insects-2757172-supplementary.PNG]
